# SARS-CoV-2 and Other Respiratory Viruses: What Does Oxidative Stress Have to Do with It?

**DOI:** 10.1155/2020/8844280

**Published:** 2020-12-21

**Authors:** Iara Grigoletto Fernandes, Cyro Alves de Brito, Vitor Manoel Silva dos Reis, Maria Notomi Sato, Nátalli Zanete Pereira

**Affiliations:** ^1^Laboratory of Medical Investigation 56, Dermatology Department, Faculty of Medicine, University of São Paulo, São Paulo, Brazil; ^2^Technical Division of Medical Biology, Immunology Center, Adolfo Lutz Institute, São Paulo, Brazil; ^3^Dermatology Department, Faculty of Medicine, University of São Paulo, São Paulo, Brazil

## Abstract

The phenomenon of oxidative stress, characterized as an imbalance in the production of reactive oxygen species and antioxidant responses, is a well-known inflammatory mechanism and constitutes an important cellular process. The relationship of viral infections, reactive species production, oxidative stress, and the antiviral response is relevant. Therefore, the aim of this review is to report studies showing how reactive oxygen species may positively or negatively affect the pathophysiology of viral infection. We focus on known respiratory viral infections, especially severe acute respiratory syndrome coronaviruses (SARS-CoVs), in an attempt to provide important information on the challenges posed by the current COVID-19 pandemic. Because antiviral therapies for severe acute respiratory syndrome coronaviruses (e.g., SARS-CoV-2) are rare, knowledge about relevant antioxidant compounds and oxidative pathways may be important for understanding viral pathogenesis and identifying possible therapeutic targets.

## 1. Introduction

### 1.1. Oxidative Stress and Reactive Oxygen Species

The concept of *oxidative stress*, an established term widely used in scientific and medical niches, was proposed in 1985 by Sies [1, 2]. Oxidative stress is a biological process that occurs naturally during metabolism and plays several roles, such as maintaining the balance between oxidant and antioxidant molecules and the homeostasis of cells, tissues, and organs [3, 4]. The main elements involved in oxidative stress are reactive oxygen species (ROS), characterized as reactive chemical species containing oxygen, such as superoxide anion (O_2_^•−^), hydrogen peroxide (H_2_O_2_), hydroxyl radical (^•^OH), and singlet oxygen (O_2_) [5].

**Figure 1:**
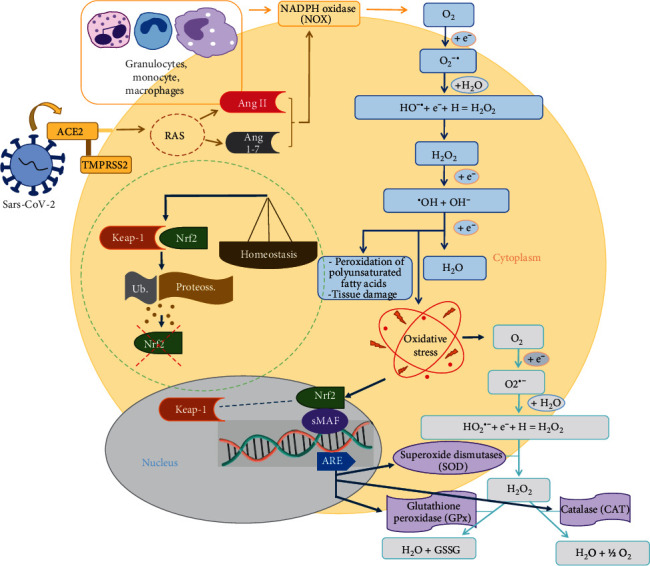
The process of reactive oxygen species formation in SARS-CoV-2 infection. The virus is able to enter the cell through the ACE2 receptor, and the serine protease TMPRSS2 is located on the membrane of some cell types. This leads to the recognition of the pathogen by a PRR, the recruitment of immune cells, and an increase in proinflammatory cytokines, thus leading to the production of reactive oxygen species and oxidative stress, representing an important immune response of the host cell. In parallel to these processes, regulation of the renin-angiotensin system (RAS) is also demonstrated, which, in this case, contributes to the increased inflammatory response and the production of ROS. NADPH oxidase (NOX), the main enzyme expressed by granulocytes and macrophages, induces the production of ROS, starting with reactive oxygen species formed from two oxygen molecules and an unpaired electron, superoxide anion (O_2_^−^). From there, the decreased proficiency of electron chain transport leads to the generation of other ROS in sequence, such as H_2_O_2_ and OH^−^. In response to the peroxidation of polyunsaturated fatty acids and tissue damage generated by oxidative stress, expression of the nuclear factor Nrf2 occurs along with the sMAF proteins, leading to the expression of antioxidant response elements (AREs) and consequently the production of antioxidant enzymes, such as superoxide dismutases (SODs), in response to exacerbated O_2_^−^ production. Glutathione peroxidases (GPXs) and catalase (CAT) act mainly by converting ROS into molecular water. Under homeostatic conditions, KEAP1 represses Nrf2 activity by marking Nrf2 for rapid degradation through the ubiquitin-proteasome system, thus preventing Nrf2 from reaching the cell nucleus and transcribing antioxidant response genes.

Through the adenosine triphosphate (ATP) synthesis process of ATP synthase, a concentration gradient of protons is established in mitochondria. In situations of cellular stress, this gradient may collapse the electron transport chain formed with electrons donated mainly from the reduced form of nicotinamide adenine dinucleotide (NADH), leading to the formation of ROS [6, 7]. In the transfer of electrons to molecular oxygen, 1 to 5% of electrons in the respiratory chain are lost, resulting mostly in the formation of superoxides (O_2_^•−^). Therefore, any molecular process that decreases the proficiency of electron chain transport may increase the production of O_2_^•−^ and consequently the formation of other ROS if more electrons are integrated into the O_2_^•−^ molecule (Figure 1) [8]. Among the ROS-producing enzymes, NADPH oxidase (NOX), an important immune mediator enzyme highly expressed in granulocytes and monocytes/macrophages, has been reported to produce ROS more than any other enzyme, including lipoxygenase [9]. However, inducible nitric oxide synthase (iNOS) is capable of producing nitric oxide (NO), a molecule involved in host defense and immune regulation [10]. Under an inflammatory state, the combination of nitric oxide and superoxide in large amounts results in the formation of peroxynitrite, which is produced by immune cells and is a strong oxidant agent [11, 12].

The most common issue caused by the overproduction of superoxide and hydrogen peroxide lies in the tissue damage that these molecules may induce, which frequently involves the generation of highly reactive hydroxyl radicals [13]. ROS produced in excessive amounts may be deleterious. However, their production is indispensable for important immunological responses against viruses and bacteria, establishing healthy cellular growth and differentiation processes and modulating the gene expression of downstream targets involved in DNA repair [14–16].

### 1.2. Antioxidant Response and Nrf2

ROS production, and thus oxidative stress, is crucial for many biological processes, including metabolism. However, it simultaneously induces an antioxidant response, primarily represented by antioxidant enzymes: superoxide dismutase (SOD), responsible for the catalysis of the conversion of superoxide radicals (O_2_^•−^) to hydrogen peroxide (H_2_O_2_), which is then converted into molecular water (H_2_O) by glutathione peroxidase (GPx) and by catalase (CAT) [17]. Importantly, three isoforms of SOD have been described in humans to date, and they are characterized by different locations: cytosolic Cu/Zn-SOD (SOD1), mitochondrial Mn-SOD (SOD2), and extracellular SOD (SOD3). These enzymes have the potential to neutralize superoxide ions by engaging in successive oxidative and reductive cycles in conjunction with transition metal ions [18]. Similarly, mammals express eight isoforms of GPx, but only GPx1, GPx2, GPx3, GPx4, and GPx6 are selenoproteins in humans [19]. Curiously, antioxidants may be recruited as direct scavengers of ROS or even as inhibitors of primary superoxide formation (and that of other individual ROS) [20].

Glutathione (GSH), a well-described intracellular antioxidant, is a potential redox regulator molecule and is responsible mostly for cellular protection from damage by free radicals, peroxides, and toxins [21]. In this context, glutathione is a potential target for investigation regarding SARS-CoV-2 infection.

Nuclear factor erythroid 2-related factor 2 (Nrf2) is a master transcription regulator of genes related to the antioxidant response [22]. Nrf2 is involved in a system associated with Kelch-like ECH-associated protein 1 (Keap-1). In this system, environmental stresses, including ROS production and electrophiles, lead to the decoupling of Keap-1. This process therefore allows Nrf2 accumulation in the cell nucleus and the formation of a heterodimer with small musculoaponeurotic fibrosarcoma (sMAF) proteins, which bind to a cis-acting element named the antioxidant responsive element (ARE), thus conferring protection against oxidative insults and cytotoxic molecules [23].

Nrf2 may prevent tissue and cell damage and decrease the production of danger-associated molecular patterns (DAMPs), which are released by necrotic cells and are immunologically important for amplifying the inflammatory response [24]. Nrf2 is an important regulator of inflammation, an essential part of innate immunity induced by infection and/or tissue injury. Protective responses are induced by Nrf2 to remove deleterious signals and initiate wound healing by the coordinated delivery of blood components (plasma and leukocytes) to the location of infection or injury caused by viruses, bacteria, or parasites [25]. However, in exacerbated inflammatory responses, the production of deleterious free radicals begins in an unbalanced way, leading to oxidative stress and the activation of different cell signaling pathways [26].

### 1.3. Oxidative Stress in Respiratory Viral Infections

Viruses are obligate intracellular parasites and hijack host cell machinery to replicate. Viral infection causes a substantial imbalance in the intracellular microenvironment, which affects, among other systems, the redox system [27]. Previous studies have already discussed the damage caused by oxidative stress in pulmonary diseases and the repercussions that develop in SARS-CoV-2 infection, showing the importance of this topic in relation to COVID-19 [28, 29], as well as apoptosis and autophagy in the same background [12].

Respiratory viruses, comprising human respiratory syncytial virus (RSV), influenza (IV), human rhinovirus (HRV), human metapneumovirus (HMPV), parainfluenza, and adenoviruses and coronaviruses (CoVs), may infect the upper and/or lower respiratory tract in humans and are the causes of the common cold (the most prevalent disease in the world). In some cases, the disease can worsen and cause other complications, such as fever and pneumonia, especially in high-risk populations, such as elderly individuals, children, and immunosuppressed patients [30].

Respiratory viruses induce ROS-generating enzymes, such as nicotinamide adenine dinucleotide phosphate oxidases (NADPH oxidase, Nox) and xanthine oxidase (XO), while creating unbalanced antioxidant levels. Murine cells infected *in vitro* with IV show a reduction in ROS production after NOx inhibition [31].

Depending on their production, ROS may play ambiguous roles during viral infections. Excess ROS, namely, superoxide and its derivatives, is the main cause of lung injury caused by influenza virus infection. Nox1 or Nox2 is also important for inducing epithelial apoptosis and lung damage by the virus [32]. Oxidative imbalance, in addition to causing tissue damage, can contribute to cell-to-cell viral transmission [33] and robust cytokine and chemokine production, leading to cytokine storms [34].

The increase in ROS production during influenza infection can activate the JNK/ERK/p38 MAPK and NF-*κ*B pathways and lead to lung damage [35]. Furthermore, an increase in nitric oxide synthase 2 (iNOS) has also been found in the lungs of patients who died from IV [33].

Increased ROS production is also observed in RSV, which causes the accumulation of lipid peroxidation products and oxidized glutathione (GSH) in the plasma of children with RSV-induced acute bronchiolitis [35, 36]. In HRV, this ROS increase is induced by enhanced O_2_ production and depleted intracellular GSH levels [37–39]. Antioxidant capacity is also suppressed in RSV-infected children and in HMPV-infected respiratory cells [40, 41].

RSV decreases Nrf2 mRNA levels in respiratory epithelial cells [42]. In addition, RSV is capable of inducing Nrf2 deacetylation and subsequent proteasomal degradation, which, in turn, leads to the downregulation of antioxidant enzyme expression [43].

The expression of antioxidants varies according to the stage of infection. In a cell culture model, during the first hours after infection, SOD1, SOD2, glutathione S-transferase (GST), CAT, and GPx are induced. With the evolution of the infection, only SOD2 continues to increase, resulting in enhanced H_2_O_2_ production, whereas other antioxidant enzymes, including those that are critical for neutralizing H_2_O_2_, are suppressed [42, 44]. The antioxidant system is also affected by IV infection, showing a decrease in SOD expression mediated by the proteasomal degradation of transcription factors that drive SOD production [45, 46]. However, no differences in SOD, CAT, or IDO expression during IV infection have been described [47]. There are, however, reports of increased SOD expression in asymptomatic IV-infected individuals [48].

Few studies have evaluated the role of the redox system in infection with HRV or human metapneumovirus (HMPV). HMPV has been shown to increase SOD2 levels and decrease SOD3, CAT, glutathione S-transferase, and peroxiredoxin 1, 3, and 6 levels [49]. HRV increases the levels and activity of SOD1 but does not affect the activity of SOD2, catalase, or GPx [50].

Antioxidant capacity is also suppressed in RSV-infected children and in HMPV-infected respiratory cells [40, 41]. In a recent study involving RSV, which promotes an infection largely related to oxidative lung injury, the degradation of Nrf2, and consequently decreased levels of antioxidant enzymes, researchers found that single-nucleotide polymorphisms (SNPs) in the catalase enzyme promoter gene provided antioxidant protection against severe RSV bronchiolitis in samples of nasopharyngeal secretions from children with the disease [51]. In agreement, *in vivo* models of RSV-infected mice treated with polyethylene glycol-conjugated catalase showed increased catalase activity and reduced H_2_O_2_ damage, neutrophil elastase, and inflammation in the airways. RSV decreases Nrf2 mRNA levels in respiratory epithelial cells [42]. In addition, RSV is capable of inducing Nrf2 deacetylation and subsequent proteasomal degradation, which in turn leads to the downregulation of antioxidant enzyme expression [43].

In viral infection, Nrf2 exerts multiple effects. Nrf2 has been described as having protective and antioxidant potential against virus-induced cell damage and viral replication of influenza A *in vitro* [52]. In contrast, Nrf2 was also described as being a negative regulator of the stimulator of IFN (STING) gene, a critical signaling molecule involved in the innate response to cytosolic nucleic acid ligands in human cells. STING is an important molecule that is also a focus of our group studying the innate immunity of HIV-infected mothers [53, 54].

### 1.4. Oxidative Stress in SARS-CoV Infections

Coronaviruses (CoVs) constitute a single-stranded RNA virus family with the largest viral genome ever described (approximately 30,000 nucleotides) [55]. Severe acute respiratory syndrome coronavirus-2 (SARS-CoV-2) is the newest member of the coronavirus family. Reported as a zoonotic virus, it likely emerged in China in the province of Hubei, where it jumped from bats and/or pangolins to humans [56]. Three months after the first reports of human infection, SARS-CoV-2 infection became a pandemic and proved to be far more lethal than its predecessors, killing more than 1.2 million people in eleven months [57].

In addition to SARS-CoV-2, six other coronaviruses can cause respiratory and intestinal diseases in humans. Four coronaviruses induce mild respiratory disease in immunosuppressed individuals or severe respiratory disease in children and elderly individuals (HCoV-NL63, HCoV-229E, HCoV-OC43, and HKU1). Two others, Middle East respiratory syndrome coronavirus (MERS-CoV) and severe acute respiratory syndrome coronavirus-1 (SARS-CoV-1), cause more severe respiratory disease and have led to major outbreaks in recent decades that combined to kill approximately 2,000 people [58, 59]. SARS-CoV-2 shares genetic similarities with SARS-CoV-1 and MERS, with 79% and 50% similarity, respectively, and all three cause respiratory disease [60].

**Figure 2:**
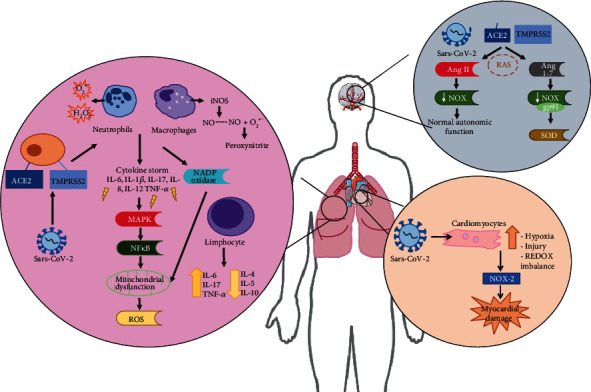
Viral entry and oxidative stress in different organs. SARS-CoV-2 can affect the lungs as well as other important organs influenced by oxidative stress. Due to the high availability of long-chain fatty acids in nerve cells in the brain and CNS, oxidative stress induced by viral entry through the ACE2 receptor and the TMPRSS2 serine protease can cause considerable damage in this system. Unbalanced RAS activation may lead to hypoxia, direct viral injury, immune-mediated damage, and ACE2 shedding. In the heart, coronaviruses cause damage, since the virus is able to directly invade cardiomyocytes, cells in the form of fibers that make up the heart muscle. In cardiac manifestations, the cytokine storm (especially represented by IL-6, TNF and IL-1*β*) plays an important role and generates oxidative stress. Such effects lead to an increase in local hypoxia, tissue injury, and REDOX imbalance. In addition, increased NOX-2 enzyme production may represent an important element considering cardiac oxidative stress and myocardial damage in COVID-19 patients. In the lungs, which are the organs most affected by infection, the virus enters alveolar and epithelial cells through the ACE2 receptor and the TMPRSS2 serine protease, which triggers the recruitment of immune cells to the infection site, leading to excessive production of proinflammatory cytokines and NOX enzyme activation. The cytokine storm can lead to the activation of MAPKs and therefore the activation of NF*κ*B, the major nuclear factor related to inflammatory responses. All of these activation pathways lead to mitochondrial dysfunction and excessive ROS production.

Coronavirus disease 2019 (COVID-19) is caused by SARS-CoV-2 infection and mainly affects the respiratory system, but COVID-19 is also capable of inducing damage to other organs. Viral transmission between humans occurs by direct contact or by contact with droplets produced by coughing or sneezing. In the lung, the virus targets type 2 alveolar cells with an affinity tenfold greater than that of SARS-CoV-1. The new coronavirus enters the host cell through the angiotensin-converting enzyme 2 (ACE2) receptor [61] (Figure 2).

Cellular invasion is also dependent on the serine protease TMPRSS2, which primes the viral spike protein [62, 63]. Cells in other organs can also express the ACE2 receptor, such as cells in the esophagus and kidneys and enterocytes in the small intestine and heart [64, 65]. ACE2 deficiency impairs endothelial function in cerebral arteries and is related to oxidative stress and aging in cerebrovascular dysfunction [66]. Angiotensin-converting enzyme 2 also has protective effects on endothelial cells through the miR-18a/Nox2/ROS pathway, as shown by ROS overproduction and the upregulation of Nox2 related to the downregulation of ACE2 [67]. Similar results have been found in renal ACE2 deficiency, which was related to increased superoxide generation [68]. Considering these findings, the relationship between the ACE2 receptor, oxidative stress, and coronaviruses should be further investigated.

After virus recognition by a pattern recognition receptor (PRR), the intracellular signaling cascade leads to type I IFN production, which in turn induces the expression of several antiviral factors that stop viral replication [54, 69]. Coronaviruses, such as SARS-CoV-1 and MERS, employ escape mechanisms to suppress the response of cytosolic and type I IFN sensors, promoting ubiquitination, inhibiting nuclear factor translocation, and/or decreasing STAT1 phosphorylation [70].

Faced with an aggressive agent, such as infection or trauma, the body may produce an exaggerated response in an attempt to locate and eliminate the damage. This process is known as systemic inflammatory response syndrome (SIRS) or, if the source is infection, sepsis [71]. Moreover, several immunological, hematological, and endocrine changes are initiated that lead to acute-phase protein release and cytokine storms. Although the objective is to eliminate the offending agent, this exacerbated response can lead to tissue damage and death [72].

The increases in several cytokines (such as IL-6, TNF, and IL-10), neutrophils, and C-reactive protein are correlated with disease severity. Increased inflammatory cytokine levels are correlated with CD4+ and CD8+ T lymphocyte decreases and decreased IFN-*γ* production [73]. This immunological profile observed in patients indicates that COVID-19, like SARS, is caused by an intense inflammatory process and that this increase in cytokine levels may be involved in disease pathogenesis [56]. A recent study proposed that the devastating production of ROS, increased formation of neutrophil extracellular traps (NETs), and, consequently, the suppression of the adaptive immune system are major causes of local or systemic tissue damage that leads to severe COVID-19 [74]. Complementing this hypothesis, another study suggested that impaired redox balance, and thus excessive ROS production, leads to red blood cell membrane peroxidation, which in turn perpetuates neutrophil activation [75].

Taking oxidative stress into consideration, granulocytes play a relevant role in viral infections, even COVID-19 [76]. Neutrophils may represent the most important cell type in this context, since they produce significant superoxide free radicals and H_2_O_2_, constituting an important mechanism in the elimination of pathogens [77]. However, the overproduction of reactive oxygen species leads to tissue damage and consequently denotes the severity of viral infections, as previously mentioned. The activation of agranulocytes, such as macrophages, leads to a respiratory burst in response to infection with SARS-CoV-2 and may also induce ROS production and therefore tissue oxidative damage, contributing to the severity of the disease and a chronic stage of infection [78].

In the lung, cytokine storms are produced mainly by highly activated macrophages and can cause complications, such as acute respiratory distress syndrome (ARDS) and respiratory and cardiac failure [79, 80]. Studies in mice infected with SARS-CoV-1 have demonstrated that cytokine storms also dampen adaptive immunity [81].

During SARS-CoV-1 infection in mice, the imbalance in antioxidant production and ROS is exacerbated [82]. Some viral proteases are able to stimulate ROS production, which in turn activates NF-*κ*B [83]. Mitogen-activated protein kinases (MAPKs) constitute a family of serine/threonine kinases that are activated (phosphorylated) during SARS-CoV-1 infection. As previously described, this activation is dependent on the cellular microenvironment state, and oxidative stress can be one of the triggers for MAPK pathway activation [27]. Moreover, *in vitro* assays have shown that SARS-CoV-1 replication is inhibited by NO in a concentration-dependent manner [84].

Although the focus of this review is pulmonary disease and its sequelae mainly caused by oxidative stress, cardiac manifestations in COVID-19 are systemically relevant and represent a result of cytokine storms in response to viral infection. Oxidative stress also plays an important role in this regard considering the direct viral invasion of cardiomyocytes, as well as typical respiratory damage from the virus that causes hypoxia and leads to redox imbalance and injury to cardiomyocytes [85]. Cardiologically, another significant consideration refers to NADPH oxidase-2 (NOX-2), which is one of the most important sources of superoxide anion in humans, appears to be increased in patients with pneumonia and is associated with an increase in troponin. These data involving the NOX-2 enzyme have been suggested as a possible cause of myocardial damage, even for COVID-19 patients [86, 87].

Several factors can contribute to disease severity, such as hypertension, asthma, heart disease, diabetes, obesity, and age [88]. The COVID-19 mortality rate is higher in elderly individuals for several reasons, such as negative ACE2 regulation; homeostatic maintenance of the renin-angiotensin system (RAS) as a negative regulator; and immunosenescent status, which consists of a loss of replicative capacity, cell apoptosis, and adverse structural changes in immune cells [89]. Although ACE2 expression is necessary for viral entry into the host cell, ACE2-knockout mice are resistant to SARS-CoV infections [90, 91], and an increase in or unchanged level of expression of this enzyme has been associated with a protective role against disease severity. In fact, ACE2 downregulation after viral entry may be involved in the pathogenesis of COVID-19. In an animal model, ACE2 depletion or inactivation after SARS-CoV infection promoted greater severity of the respiratory syndrome than that observed in wild-type animals. The loss of ACE2 led to increased vascular permeability, lung edema, and neutrophil accumulation. However, when treated with catalytically active recombinant ACE2 protein, these symptoms were ameliorated [92].

ACE2 is a component of the RAS, which regulates blood pressure as well as inflammation and oxidative stress [93, 94]. RAS regulation is initiated when angiotensinogen (Ang), produced by the liver and adipocytes, is cleaved by renin to form angiotensin I. The cleaved form may follow two main axes: the first is dependent on ACE and leads to Ang II formation, and the second depends on both ACE and ACE2 and leads to Ang1-7 formation. Ang II may act by binding to two receptors, the Ang II type 1 receptor (AT1R) and Ang II type 2 receptor (AT2R). AT1R signaling induces mechanisms that increase blood pressure and inflammation, while AT2R induction has the opposite effect. On the other hand, Ang1-7 binds to the receptors AT2R, MasR, and MRgD, resulting in antagonistic effects on the Ang II/AT1R axis [95]. The relationship between the RAS, mainly the ACE/Ang II/AT1R axis, and ROS production has been described. Ang II may indirectly increase ROS production by the induction of proinflammatory cytokines, such as TNF-*α*, IL-1*β*, and IL-6. Furthermore, Ang II binding to AT1R promotes ROS production by NOX protein activation through the mediators protein kinase C (PKC) and Src kinases. In turn, ROS cause mitochondrial dysfunction and, consequently, further ROS production [94, 96]. Ang II also inhibits antioxidant molecules. The treatment of rat cardiac fibroblasts with Ang II increased the production of superoxide ions and decreased the activity of Mn-SOD and Cu/Zn-SOD [97]. Supporting these findings, fimasartan, an AT1R blocker, inhibits Nox expression and increases the expression of Nrf2 and antioxidant enzymes, such as CuSOD, Mn-SOD, and catalase [98].

Since the global spread of COVID-19, several reports about neurological symptoms have emerged [99, 100]. The detection of SARS-CoV [101] and SARS-CoV-2 [102] in cerebrospinal fluid has confirmed that coronaviruses can invade the central nervous system (CNS). Some mechanisms have been proposed to explain CNS damage by SARS-CoV-2, such as hypoxia, direct viral injury, immune-mediated damage, and ACE2 shedding [103]. It is possible that oxidative stress is elicited at least in the proposed mechanisms of immune-mediated damage and ACE2 shedding. In fact, because of the large amounts of polyunsaturated fatty acids, the brain is particularly vulnerable to ROS [104], and oxidative stress is suggested to be involved in several neurodegenerative and neuropsychiatric disorders, ranging from depression to Alzheimer's disease [105]. Again, imbalanced RAS activation in the brain may be related to encephalopathy in COVID-19. The ACE2/Ang1-7/Mas axis is reported to confer protection against cerebrovascular diseases, such as ischemic stroke, eliciting antithrombotic, anti-inflammatory, and antioxidative effects [106]. In an experimental model of *Ace2*-knockout mice infused with Ang II, gene therapy with an adenovirus vector expressing ACE2 in the hypothalamus was able to reduce NOX activity and normalize autonomic function [107]. Furthermore, another study showed that treatment with Ang1-7 attenuated neuronal apoptosis, which was accompanied by elevated SOD activity and reduced NOX gp91^phox^ levels in the brains of spontaneously hypertensive rats [108].

### 1.5. Therapeutic Strategies

Cytokine storms lead to leukocyte accumulation and activation in the lungs; thus, ROS and proteases are produced in large amounts, leading to damage to the capillary endothelium and alveolar epithelium [109]. Many studies have shown that natural products, vitamins, and compounds are important agents with anti-inflammatory and antioxidant properties that might provide promising treatment and/or prevention of the progression of COVID-19 [110–112].

In the immune system, vitamins, such as C, D, and E, seem to play an important role against SARS-CoV2 infection. Vitamin D (25-hydroxyvitamin D (25(OH)VD) comprises a number of fat-soluble secosteroids and has been increasingly described due to its anti-inflammatory and epigenetic regulator potential [113–115]. In immune cells, vitamin D is able to regulate effector T cell differentiation by modulating antigen-presenting dendritic cells (DCs) and by decreasing the synthesis of IL-12, a cytokine that promotes Th1 cell responses [116]. Additionally, this component showed the ability to differentiate naive T cells into the Th17 cell type [117], as well as potential to stimulate the production of IFN-I and cathelicidins and defensins (AMPs), which are molecules with important antiviral action in this context [118, 119]. Previous studies have shown positive associations between vitamin D deficiency and mortality in subjects with severe forms of pneumonia [120] and with the severity of COVID-19 [121], especially in elderly individuals [122, 123]. In this regard, vitamin D supplementation promotes binding of the SARS-CoV-2 cell entry receptor ACE2 to AGTR1 (angiotensin II receptor type 1), thus creating fewer opportunities for the virus to attach to ACE2 and enter the cell [124]. Considering oxidative stress, vitamin D also has relevance. The antioxidant enzyme glutathione (GSH) is required to maintain circulating levels of 25-hydroxyvitamin D (25(OH)VD) [125]. Therefore, viral infection-mediated excess oxidative stress might be considered a relevant target in this therapeutic approach.

Vitamin E is a lipid-soluble compound with relevant antioxidant properties and has eight distinct groups, known as *α*-, *β*-, *γ*-, and *δ*-tocopherols and *α*-, *β*-, *γ*-, and *δ*-tocotrienols [126]. The most biologically available isoform is *α*-tocopherol, which is found in hazelnuts, peanuts, and avocado, among other foods. This micronutrient is described as an effective antioxidant considering its capacity to counteract free radicals and ROS by donating a hydrogen ion from its chromanol ring [127]. One of the most damaging effects of ROS is lipid peroxidation of the cell membrane, and vitamin E plays an important role in this regard by protecting polyunsaturated fatty acids in the membrane from oxidation [128, 129]. Immunologically, *α*-tocopherol plays many roles in different cell types. Considering the importance of pulmonary diseases in coronaviruses, the immunomodulatory effects highlighted are decreased production of prostaglandin E2 (PGE2), cyclooxygenase 2 COX2, and nitric oxide (NO) by macrophages; increased T cell proliferation and natural killer cell (NK) activity; and the intensification of the antibody response by B cells [130].

Moreover, a study involving a murine model showed that vitamin E (*α*-tocopherol) in combination with oseltamivir (neuraminidase inhibitor) reduced the mortality rate of infection with influenza virus, decreased infectious virus content when analyzing lung parameters and showed a marked diminishment in the lung index and pathology [131]. L-ascorbic acid (vitamin C) showed antiviral immune responses against IV in a mouse model through increased production of IFN-*α*/*β* [132].

Resveratrol is a polyphenolic compound found in red wine, grapes, cocoa, and other foods. It presents anti-inflammatory properties by interfering with immune cell regulation and proinflammatory cytokine synthesis. In addition, resveratrol has a protective role against several diseases, such as cancer, cardiovascular disease, and respiratory illness [133–136]. In a mouse model, resveratrol administration resulted in ACE2 dysregulation and abdominal aortic aneurysm growth inhibition [137]. Moreover, resveratrol treatment resulted in significantly improved survival and decreased pulmonary viral titers in IV-infected mice [138].

GSH has also shown promise in assays conducted with mice *in vitro*. The addition of GSH to drinking water decreased the viral titers in the lung and trachea in animals infected with IV [139]. Glutathione assumes a protective role against peroxynitrite-mediated DNA damage during acute inflammation, supporting a potential therapeutic strategy in severe COVID-19 cases [140]. Ebselen, an organoselenium compound, mimics glutathione peroxidase and peroxiredoxin enzyme activity [141]. This compound protects the lung against oxidative stress-induced lung inflammation *in vivo*, mainly caused by the enhanced presence of neutrophils and macrophages, proteolytic burden, and IL-17 expression in bronchoalveolar lavage fluid [142]. Ebselen uses a glutathione peroxidase-1 (GPx1) mimetic to reduce influenza A virus-induced lung inflammation [143]. Given that GSH is important for immune responses due to the activation of antioxidant mechanisms and optimal functioning of lymphocytes and other immune cells [144], natural compounds that activate the Nrf2-antioxidant response element (ARE) pathway and thus glutathione and other antioxidant elements may be promising targets.

Naringenin is a flavonoid abundantly found in citrus fruits and has shown prominent therapeutic potential in a variety of diseases, especially due to its anti-inflammatory and antioxidant activities [145]. A study using lipopolysaccharide- (LPS-) induced injury in a normal human bronchial epithelium model indicated that naringenin was able to attenuate mitogen-activated protein kinase (MAPK) activation by inhibiting the phosphorylation of ERK1/2, c-Jun NH(2)-terminal kinase (JNK), and p38 MAPK. These findings suggest that naringenin reduces secretion of the proinflammatory cytokines TNF-*α* and IL-6 and mRNA expression, likely by blocking activation of the NF-*κ*B and MAPK pathways [146]. Furthermore, naringenin is capable of activating Nrf2 and consequently inducing the production of antioxidant enzymes, including GPX [147].

Most studies involving antioxidant therapeutic approaches are directed against IV and HSRV in cell or mouse models. N-acetylcysteine (NAC), an analog and precursor of reduced glutathione, has shown promise against the effects of IV infection. Long-term treatment (6 months) with NAC resulted in a significant decrease in the frequency of influenza-like episodes, the severity of the symptoms, and the length of time confined in bed [148]. In another *in vitro* assay, adding zinc to the culture medium after RSV infection led to significant inhibition of RSV titers [149].

Another antioxidant with antiviral activity is CAT. In mice, CAT was able to suppress the inflammatory response by promoting a protective role against pneumonia [150]. The survival time and rates of mice with H1N1-induced pneumonia were increased by treatment with recombinant human CAT [151].

Thioredoxin (Trx) is a ubiquitous thiol oxidoreductase system that has different isoforms: thioredoxin, thioredoxin reductase, and NADPH. This system plays a role in a variety of biological processes related to defense against oxidative stress [152]. Trx is widely expressed in type II pneumocytes, macrophages, and bronchial epithelial cells and may be regulated by Nrf2 and thus express AREs [153]. In patients with acute lung injury, extracellular thioredoxin levels were increased, indicating acute lung injury [154]. A study involving a murine model of influenza pneumonia showed that Trx-1 significantly enhanced the survival rate and attenuated lung histological changes, suggesting a pharmacological strategy for severe influenza virus infection [155]. A recombinant human serum albumin-thioredoxin 1 (Trx) fusion protein has also been demonstrated as an interesting therapeutic approach by inhibiting inflammatory cell responses and suppressing the overproduction of NO in the lung [156].

Protein disulfide isomerases (PDIs) constitute a superfamily of redox chaperones that participate in important cellular redox state processes, such as the modulation of cellular oxidative stress mediating homeostasis of the antioxidant glutathione [157], modulation of endoplasmic reticulum stress, the unfolded protein response, communication between endoplasmic reticulum and mitochondria, and the balance between cell proliferation and apoptosis [158]. A pulmonary fibrosis study showed that a domain of PDI (TXNDC5) was highly upregulated in patients with idiopathic pulmonary fibrosis as well as a mouse model of this injury, suggesting that this protein could be a novel therapeutic target in the treatment of pulmonary fibrosis [159]. There are currently no studies showing the effects of PDIs on COVID-19. However, the deletion of PDIA3 (a member of the PDI family) in mice is associated with decreased viral burden and proinflammatory responses from lung epithelial cells in influenza A virus infection [160].

Our suggestion, therefore, is to evaluate foods that contain these antioxidants and vitamins as both nutrients and quality agents for preventing severe SARS-CoV-2 infection, since prevention through balanced eating and healthy habits is more important than therapeutic treatment. Considering critically ill patients, we consider a potential therapeutic strategy for COVID-19 to include compounds that have anti-inflammatory and antioxidant actions, highlighting those capable of decreasing the effects of the cytokine storm, activating Nrf2-ARE and blocking activation of the NF-*κ*B pathway, as well as presenting antiviral activity by enhancing the production of IFN-I. Nevertheless, taking into consideration that some compounds may increase the production of antibodies, their use or vitamin supplementation may be a strategy to enhance vaccine efficacy.

## 2. Conclusion

Respiratory viruses lead to many deaths and can spread worldwide. In addition to pronounced inflammation, they also cause changes in the redox system. Little is known about the mechanisms involved in this imbalance, but oxidative stress likely contributes to the increased inflammation and tissue damage caused by the infection. The roles of antioxidants may be instrumental in balancing the expression of ROS and regulating inflammation. However, tests to prove the effectiveness of antioxidants are limited to *in vitro* and animal models. Clinical studies are required to try to restore the oxidative system in humans with viral infections.

## References

[1] Sies H. (1985). Oxidative stress: introductory remarks oxidative stress. *New York Academic Journal*.

[2] Sies H. (2015). Oxidative stress: a concept in redox biology and medicine. *Redox Biology*.

[3] Proskurnina E. V., Izmailov D. Y., Sozarukova M. M., Zhuravleva T. A., Leneva I. A., Poromov A. A. (2020). Antioxidant potential of antiviral drug Umifenovir. *Molecules*.

[4] Imlay J. A. (2019). Where in the world do bacteria experience oxidative stress?. *Environmental Microbiology*.

[5] Hayyan M., Hashim M. A., AlNashef I. M. (2016). Superoxide ion: generation and chemical implications. *Chemical Reviews*.

[6] Klingenberg M. (1980). The ADP-ATP translocation in mitochondria, a membrane potential controlled transport. *The Journal of Membrane Biology*.

[7] Zhang Y., Marcillat O., Giulivi C., Ernster L., Davies K. (1990). The oxidative inactivation of mitochondrial electron transport chain components and ATPase. *Journal of Biological Chemistry*.

[8] Green D. R., Reed J. C. (1998). Mitochondria and apoptosis. *Science*.

[9] Cadenas E., Davies K. J. (2000). Mitochondrial free radical generation, oxidative stress, and aging^1^. *Free Radical Biology and Medicine*.

[10] Uehara E. U., Shida Bde S., de Brito C. A. (2015). Role of nitric oxide in immune responses against viruses: beyond microbicidal activity. *Inflammation Research*.

[11] Koppenol W., Moreno J., Pryor W. A., Ischiropoulos H., Beckman J. (1992). Peroxynitrite, a cloaked oxidant formed by nitric oxide and superoxide. *Chemical Research in Toxicology*.

[12] Fakhri S., Nouri Z., Moradi S. Z., Farzaei M. H. (2020). Astaxanthin, COVID-19 and immune response: focus on oxidative stress, apoptosis and autophagy. *Phytotherapy Research*.

[13] Halliwell B. (1989). Oxidants and the central nervous system: some fundamental questions. Is oxidant damage relevant to Parkinson's disease, Alzheimer's disease, traumatic injury or stroke?. *Acta Neurologica Scandinavica*.

[14] Imlay J. A. (2013). The molecular mechanisms and physiological consequences of oxidative stress: lessons from a model bacterium. *Nature Reviews Microbiology*.

[15] Elbim C., Pillet S., Prevost M. H. (1999). Redox and activation status of monocytes from human immunodeficiency virus-infected patients: relationship with viral load. *Journal of Virology*.

[16] Lander H. M. (1997). An essential role for free radicals and derived species in signal transduction. *The FASEB Journal*.

[17] Balmus I. M., Ciobica A., Antioch I., Dobrin R., Timofte D. (2016). Oxidative stress implications in the affective disorders: main biomarkers, animal models relevance, genetic perspectives, and antioxidant approaches. *Oxidative medicine and cellular longevity*.

[18] Kurutas E. B. (2015). The importance of antioxidants which play the role in cellular response against oxidative/nitrosative stress: current state. *Nutrition Journal*.

[19] Kryukov G. V., Castellano S., Novoselov S. V. (2003). Characterization of mammalian selenoproteomes. *Science*.

[20] Plecitá-Hlavatá L., Engstová H., Ježek J. (2019). Potential of mitochondria-targeted antioxidants to prevent oxidative stress in pancreatic *β*-cells. *Oxidative medicine and cellular longevity*.

[21] Wu J. H., Batist G. (2013). Glutathione and glutathione analogues; therapeutic potentials. *Biochimica et Biophysica Acta*.

[22] He F., Antonucci L., Karin M. (2020). NRF2 as a regulator of cell metabolism and inflammation in cancer. *Carcinogenesis*.

[23] Kobayashi E., Suzuki T., Yamamoto M. (2013). Roles nrf2 plays in myeloid cells and related disorders. *Oxidative medicine and cellular longevity*.

[24] Tang W., Jiang Y.-F., Ponnusamy M., Diallo M. (2014). Role of Nrf2 in chronic liver disease. *World Journal of Gastroenterology*.

[25] Medzhitov R. (2008). Origin and physiological roles of inflammation. *Nature*.

[26] Mohan S., Gupta D. (2018). Crosstalk of toll-like receptors signaling and Nrf2 pathway for regulation of inflammation. *Biomedicine & Pharmacotherapy*.

[27] Khomich O. A., Kochetkov S. N., Bartosch B., Ivanov A. V. (2018). Redox biology of respiratory viral infections. *Viruses*.

[28] Cecchini R., Cecchini A. L. (2020). SARS-CoV-2 infection pathogenesis is related to oxidative stress as a response to aggression. *Medical hypotheses*.

[29] Derouiche S. (2020). Oxidative stress associated with SARS-Cov-2 (COVID-19) increases the severity of the lung disease-a systematic review. *Journal of Infectious Diseases and Epidemiology*.

[30] Liu M., Chen F., Liu T., Chen F., Liu S., Yang J. (2017). The role of oxidative stress in influenza virus infection. *Microbes and Infection*.

[31] Amatore D., Sgarbanti R., Aquilano K. (2015). Influenza virus replication in lung epithelial cells depends on redox-sensitive pathways activated by NOX4-derived ROS. *Cellular Microbiology*.

[32] Lim J. Y., Oh E., Kim Y. (2014). Enhanced oxidative damage to DNA, lipids, and proteins and levels of some antioxidant enzymes, cytokines, and heat shock proteins in patients infected with influenza H1N1 virus. *Acta Virologica*.

[33] Nin N., Sanchez-Rodriguez C., Ver LS C. P. (2012). Lung histopathological findings in fatal pandemic influenza A (H1N1). *Medicina Intensiva*.

[34] Hennet T., Peterhans E., Stocker R. (1992). Alterations in antioxidant defences in lung and liver of mice infected with influenza A virus. *The Journal of General Virology*.

[35] Martínez I., García-Carpizo V., Guijarro T. (2016). Induction of DNA double-strand breaks and cellular senescence by human respiratory syncytial virus. *Virulence*.

[36] Moreno‐Solís G., dela Torre‐Aguilar M. J., Torres‐Borrego J. (2017). Oxidative stress and inflamatory plasma biomarkers in respiratory syncytial virus bronchiolitis. *The Clinical Respiratory Journal*.

[37] Biagioli M. C., Kaul P., Singh I., Turner R. B. (1999). The role of oxidative stress in rhinovirus induced elaboration of IL-8 by respiratory epithelial cells. *Free Radical Biology & Medicine*.

[38] Papi A., Contoli M., Gasparini P. (2008). Role of xanthine oxidase activation and reduced glutathione depletion in rhinovirus induction of inflammation in respiratory epithelial cells. *The Journal of Biological Chemistry*.

[39] Kaul P., Biagioli M. C., Singh I., Turner R. B. (2000). Rhinovirus-induced oxidative stress and interleukin-8 elaboration involves p47-phox but is independent of attachment to intercellular adhesion molecule-1 and viral replication. *The Journal of Infectious Diseases*.

[40] Hosakote Y. M., Jantzi P. D., Esham D. L. (2011). Viral-mediated inhibition of antioxidant enzymes contributes to the pathogenesis of severe respiratory syncytial virus bronchiolitis. *American Journal of Respiratory and Critical Care Medicine*.

[41] Mochizuki H., Todokoro M., Arakawa H. (2009). RS virus-induced inflammation and the intracellular glutathione redox state in cultured human airway epithelial cells. *Inflammation*.

[42] Hosakote Y. M., Liu T., Castro S. M., Garofalo R. P., Casola A. (2009). Respiratory syncytial virus induces oxidative stress by modulating antioxidant enzymes. *American Journal of Respiratory Cell and Molecular Biology*.

[43] Komaravelli N., Tian B., Ivanciuc T. (2015). Respiratory syncytial virus infection down-regulates antioxidant enzyme expression by triggering deacetylation-proteasomal degradation of Nrf2. *Free Radical Biology and Medicine*.

[44] Garofalo R. P., Kolli D., Casola A. (2013). Respiratory syncytial virus infection: mechanisms of redox control and novel therapeutic opportunities. *Antioxidants & Redox Signaling*.

[45] Erkekoğlu P., Aşçı A., Ceyhan M. (2013). Selenium levels, selenoenzyme activities and oxidant/antioxidant parameters in H1N1-infected children. *The Turkish Journal of Pediatrics*.

[46] Lin X., Wang R., Zou W. (2016). The influenza virus H5N1 infection can induce ROS production for viral replication and host cell death in A549 cells modulated by human Cu/Zn superoxide dismutase (SOD1) overexpression. *Viruses*.

[47] Jacoby D. B., Choi A. M. (1994). Influenza virus induces expression of antioxidant genes in human epithelial cells. *Free Radical Biology & Medicine*.

[48] Huang Y., Zaas A. K., Rao A. (2011). Temporal dynamics of host molecular responses differentiate symptomatic and asymptomatic influenza A infection. *PLoS Genetics*.

[49] Bao X., Sinha M., Liu T. (2008). Identification of human metapneumovirus-induced gene networks in airway epithelial cells by microarray analysis. *Virology*.

[50] Kaul P., Singh I., Turner R. B. (2002). Effect of rhinovirus challenge on antioxidant enzymes in respiratory epithelial cells. *Free Radical Research*.

[51] Chambliss J. M., Ansar M., Kelley J. P., Spratt H., Garofalo R. P., Casola A. (2020). A polymorphism in the catalase gene promoter confers protection against severe RSV bronchiolitis. *Viruses*.

[52] Kosmider B., Messier E. M., Janssen W. J. (2012). Nrf2 protects human alveolar epithelial cells against injury induced by influenza A virus. *Respiratory research*.

[53] Olagnier D., Brandtoft A. M., Gunderstofte C. (2018). Nrf2 negatively regulates STING indicating a link between antiviral sensing and metabolic reprogramming. *Nature Communications*.

[54] Pereira N. Z., Cardoso E. C., da Silva Oliveira L. M. (2013). Upregulation of innate antiviral restricting factor expression in the cord blood and decidual tissue of HIV-infected mothers. *PloS one*.

[55] Chan J. F., Kok K. H., Zhu Z. (2020). Genomic characterization of the 2019 novel human-pathogenic coronavirus isolated from a patient with atypical pneumonia after visiting Wuhan. *Emerging microbes & infections*.

[56] Huang C., Wang Y., Li X. (2020). Clinical features of patients infected with 2019 novel coronavirus in Wuhan, China. *Lancet*.

[57] Organization WH (2020). *Coronavirus disease (COVID-19) pandemic*.

[58] Su S., Wong G., Shi W. (2016). Epidemiology, genetic recombination, and pathogenesis of coronaviruses. *Trends in Microbiology*.

[59] Ksiazek T. G., Erdman D., Goldsmith C. S. (2003). A novel coronavirus associated with severe acute respiratory syndrome. *The New England Journal of Medicine*.

[60] Lu R., Zhao X., Li J. (2020). Genomic characterisation and epidemiology of 2019 novel coronavirus: implications for virus origins and receptor binding. *Lancet*.

[61] Zhou P., Yang X. L., Wang X. G. (2020). A pneumonia outbreak associated with a new coronavirus of probable bat origin. *Nature*.

[62] Wrapp D., Wang N., Corbett K. S. (2020). Cryo-EM structure of the 2019-nCoV spike in the prefusion conformation. *Science*.

[63] Hoffmann M., Kleine-Weber H., Schroeder S. (2020). SARS-CoV-2 cell entry depends on ACE2 and TMPRSS2 and is blocked by a clinically proven protease inhibitor. *Cell*.

[64] Zou X., Chen K., Zou J., Han P., Hao J., Han Z. (2020). Single-cell RNA-seq data analysis on the receptor ACE2 expression reveals the potential risk of different human organs vulnerable to 2019-nCoV infection. *Frontiers in Medicine*.

[65] Hamming I., Timens W., Bulthuis M. L., Lely A. T., Navis G., van Goor H. (2004). Tissue distribution of ACE2 protein, the functional receptor for SARS coronavirus. A first step in understanding SARS pathogenesis. *The Journal of Pathology*.

[66] Silva R. A., Chu Y., Miller J. D. (2012). Impact of ACE2 deficiency and oxidative stress on cerebrovascular function with aging. *Stroke*.

[67] Zhang C., Wang J., Ma X. (2018). ACE2-EPC-EXs protect ageing ECs against hypoxia/reoxygenation-induced injury through the miR-18a/Nox2/ROS pathway. *Journal of cellular and molecular medicine*.

[68] Jin H.-Y., Chen L.-J., Zhang Z.-Z. (2015). Deletion of angiotensin-converting enzyme 2 exacerbates renal inflammation and injury in apolipoprotein E-deficient mice through modulation of the nephrin and TNF-alpha-TNFRSF1A signaling. *Journal of Translational Medicine*.

[69] de Brito C. A., Goldoni A. L., Sato M. N. (2009). Immune adjuvants in early life: targeting the innate immune system to overcome impaired adaptive response. *Immunotherapy*.

[70] Kindler E., Thiel V., Weber F. (2016). Interaction of SARS and MERS coronaviruses with the antiviral interferon response. *Advances in Virus Research*.

[71] Bone R. C., Balk R. A., Cerra F. B. (1992). Definitions for sepsis and organ failure and guidelines for the use of innovative therapies in sepsis. The ACCP/SCCM Consensus Conference Committee. American College of Chest Physicians/Society of Critical Care Medicine. *Chest*.

[72] Toliver-Kinsky T., Kobayashi M., Suzuki F., Sherwood E. R. (2018). The systemic inflammatory response syndrome. *Total Burn Care*.

[73] Chen G., Wu D., Guo W. (2020). Clinical and immunologic features in severe and moderate coronavirus disease 2019. *The Journal of Clinical Investigation*.

[74] Schönrich G., Raftery M. J., Samstag Y. (2020). Devilishly radical NETwork in COVID-19: oxidative stress, neutrophil extracellular traps (NETs), and T cell suppression. *Advances in biological regulation*.

[75] Laforge M., Elbim C., Frère C. (2020). Tissue damage from neutrophil-induced oxidative stress in COVID-19. *Nature Reviews. Immunology*.

[76] Salib C., Teruya-Feldstein J. (2020). Hypersegmented granulocytes and COVID-19 infection. *Blood*.

[77] Naumenko V., Turk M., Jenne C. N., Kim S.-J. (2018). Neutrophils in viral infection. *Cell and tissue research*.

[78] Zhang H., Liu H., Zhou L., Yuen J., Forman H. J. (2017). Temporal changes in glutathione biosynthesis during the lipopolysaccharide-induced inflammatory response of THP-1 macrophages. *Free Radical Biology and Medicine*.

[79] Mahallawi W. H., Khabour OF, Zhang Q., Makhdoum H. M., Suliman B. A. (2018). MERS-CoV infection in humans is associated with a pro-inflammatory Th1 and Th17 cytokine profile. *Cytokine*.

[80] Liao M., Liu Y., Yuan J. (2020). The landscape of lung bronchoalveolar immune cells in COVID-19 revealed by single-cell RNA sequencing. *medRxiv*.

[81] Channappanavar R., Fehr A. R., Vijay R. (2016). Dysregulated type I interferon and inflammatory monocyte-macrophage responses cause lethal pneumonia in SARS-CoV-infected mice. *Cell Host & Microbe*.

[82] van den Brand J. M., Haagmans B. L., van Riel D., Osterhaus A. D., Kuiken T. (2014). The pathology and pathogenesis of experimental severe acute respiratory syndrome and influenza in animal models. *Journal of Comparative Pathology*.

[83] Lin C. W., Lin K. H., Hsieh T. H., Shiu S. Y., Li J. Y. (2006). Severe acute respiratory syndrome coronavirus 3C-like protease-induced apoptosis. *FEMS Immunology and Medical Microbiology*.

[84] Akerström S., Mousavi-Jazi M., Klingström J., Leijon M., Lundkvist A., Mirazimi A. (2005). Nitric oxide inhibits the replication cycle of severe acute respiratory syndrome coronavirus. *Journal of Virology*.

[85] Tahir F., Bin Arif T., Ahmed J., Malik F., Khalid M. (2020). Cardiac manifestations of coronavirus disease 2019 (COVID-19): a comprehensive review. *Cureus*.

[86] Violi F., Carnevale R., Loffredo L., Pignatelli P., Gallin J. I. (2017). NADPH oxidase-2 and atherothrombosis: insight from chronic granulomatous disease. *Arteriosclerosis, Thrombosis, and Vascular Biology*.

[87] Loffredo L., Violi F. (2020). COVID-19 and cardiovascular injury: a role for oxidative stress and antioxidant treatment?. *International Journal of Cardiology*.

[88] Alberca R., Oliveira L., Branco A., Pereira N., Sato M. (2020). Obesity as a risk factor for COVID-19: an overview. *Critical Reviews In Food Science And Nutrition*.

[89] Jia G., Aroor A. R., Jia C., Sowers J. R. (2019). Endothelial cell senescence in aging-related vascular dysfunction. *Biochimica et Biophysica Acta - Molecular Basis of Disease*.

[90] Imai Y., Kuba K., Ohto-Nakanishi T., Penninger J. M. (2010). Angiotensin-converting enzyme 2 (ACE2) in disease pathogenesis. *Circulation Journal*.

[91] Kuba K., Imai Y., Rao S. (2005). A crucial role of angiotensin converting enzyme 2 (ACE2) in SARS coronavirus-induced lung injury. *Nature Medicine*.

[92] Imai Y., Kuba K., Rao S. (2005). Angiotensin-converting enzyme 2 protects from severe acute lung failure. *Nature*.

[93] Rodrigues Prestes T. R., Rocha N. P., Miranda A. S., Teixeira A. L., Simoes-E-Silva A. C. (2017). The anti-inflammatory potential of ACE2/angiotensin-(1-7)/mas receptor axis: evidence from basic and clinical research. *Current Drug Targets*.

[94] Ramalingam L., Menikdiwela K., Le Mieux M. (2017). The renin angiotensin system, oxidative stress and mitochondrial function in obesity and insulin resistance. *Biochimica et Biophysica Acta - Molecular Basis of Disease*.

[95] Azushima K., Morisawa N., Tamura K., Nishiyama A. (2020). Recent research advances in renin-angiotensin-aldosterone system receptors. *Current Hypertension Reports*.

[96] Doughan A. K., Harrison D. G., Dikalov S. I. (2008). Molecular mechanisms of angiotensin II-mediated mitochondrial dysfunction: linking mitochondrial oxidative damage and vascular endothelial dysfunction. *Circulation Research*.

[97] Lijnen P. J., van Pelt J. F., Fagard R. H. (2010). Downregulation of manganese superoxide dismutase by angiotensin II in cardiac fibroblasts of rats: association with oxidative stress in myocardium. *American Journal of Hypertension*.

[98] Kim S., Kim S. J., Yoon H. E. (2015). Fimasartan, a novel angiotensin-receptor blocker, protects against renal inflammation and fibrosis in mice with unilateral ureteral obstruction: the possible role of Nrf2. *International Journal of Medical Sciences*.

[99] Helms J., Kremer S., Merdji H. (2020). Neurologic features in severe SARS-CoV-2 infection. *The New England Journal of Medicine*.

[100] Ferrarese C., Silani V., Priori A. (2020). An Italian multicenter retrospective-prospective observational study on neurological manifestations of COVID-19 (NEUROCOVID). *Neurological Sciences*.

[101] Lau K. K., Yu W. C., Chu C. M., Lau S. T., Sheng B., Yuen K. Y. (2004). Possible central nervous system infection by SARS coronavirus. *Emerging Infectious Diseases*.

[102] Moriguchi N., Yamamoto S., Isokawa S., Andou A., Miyata H. (2006). Granulocyte functions and changes in ability with age in newborns; report no. 1: flow cytometric analysis of granulocyte functions in whole blood. *Pediatrics International*.

[103] Wu Y., Xu X., Chen Z. (2020). Nervous system involvement after infection with COVID-19 and other coronaviruses. *Brain, Behavior, and Immunity*.

[104] Rodrigo R., Fernández-Gajardo R., Gutiérrez R. (2013). Oxidative stress and pathophysiology of ischemic stroke: novel therapeutic opportunities. *CNS & Neurological Disorders Drug Targets*.

[105] Salim S. (2017). Oxidative stress and the central nervous system. *The Journal of Pharmacology and Experimental Therapeutics*.

[106] Jiang T., Gao L., Lu J., Zhang Y. D. (2013). ACE2-Ang-(1-7)-Mas axis in brain: a potential target for prevention and treatment of ischemic stroke. *Current Neuropharmacology*.

[107] Xia H., Suda S., Bindom S. (2011). ACE2-mediated reduction of oxidative stress in the central nervous system is associated with improvement of autonomic function. *PLoS One*.

[108] Jiang T., Gao L., Shi J., Lu J., Wang Y., Zhang Y. (2013). Angiotensin-(1-7) modulates renin-angiotensin system associated with reducing oxidative stress and attenuating neuronal apoptosis in the brain of hypertensive rats. *Pharmacological Research*.

[109] Sarzi-Puttini P., Giorgi V., Sirotti S. (2020). COVID-19, cytokines and immunosuppression: what can we learn from severe acute respiratory syndrome?. *Clinical and Experimental Rheumatology*.

[110] Azab A., Nassar A., Azab A. N. (2016). Anti-inflammatory activity of natural products. *Molecules*.

[111] Yang R., Yuan B. C., Ma Y. S., Zhou S., Liu Y. (2017). The anti-inflammatory activity of licorice, a widely used Chinese herb. *Pharmaceutical Biology*.

[112] Zhang X. L., Guo Y. S., Wang C. H. (2014). Phenolic compounds from Origanum vulgare and their antioxidant and antiviral activities. *Food Chemistry*.

[113] Yin K., Agrawal D. K. (2014). Vitamin D and inflammatory diseases. *Journal of inflammation research*.

[114] Panfili F., Roversi M., D’Argenio P., Rossi P., Cappa M., Fintini D. (2020). Possible role of vitamin D in Covid-19 infection in pediatric population. *Journal of endocrinological investigation*.

[115] Carlberg C. (2019). Vitamin D signaling in the context of innate immunity: focus on human monocytes. *Frontiers in immunology*.

[116] D'Ambrosio D., Cippitelli M., Cocciolo M. G. (1998). Inhibition of IL-12 production by 1, 25-dihydroxyvitamin D3. Involvement of NF-kappaB downregulation in transcriptional repression of the p40 gene. *The Journal of clinical investigation*.

[117] Daniel C., Sartory N. A., Zahn N., Radeke H. H., Stein J. M. (2008). Immune modulatory treatment of trinitrobenzene sulfonic acid colitis with calcitriol is associated with a change of a T helper (Th) 1/Th17 to a Th2 and regulatory T cell profile. *Journal of Pharmacology and Experimental Therapeutics*.

[118] Bhalla A. K., Amento E. P., Krane S. M. (1986). Differential effects of 1, 25-dihydroxyvitamin D3 on human lymphocytes and monocyte/macrophages: inhibition of interleukin-2 and augmentation of interleukin-1 production. *Cellular immunology*.

[119] Beard J. A., Bearden A., Striker R. (2011). Vitamin D and the anti-viral state. *Journal of Clinical Virology*.

[120] Remmelts H. H., van de Garde E. M., Meijvis S. C. (2012). Addition of vitamin D status to prognostic scores improves the prediction of outcome in community-acquired pneumonia. *Clinical infectious diseases*.

[121] Mercola J., Grant W. B., Wagner C. L. (2020). Evidence regarding vitamin D and risk of COVID-19 and its severity. *Nutrients*.

[122] Pereira M., Dantas Damascena A., Galvão Azevedo L. M., de Almeida O. T., da Mota Santana J. (2020). Vitamin D deficiency aggravates COVID-19: systematic review and meta-analysis. *Critical Reviews in Food Science and Nutrition*.

[123] Ferrari D., Locatelli M., Briguglio M., Lombardi G. (2020). Is there a link between vitamin D status, SARS-CoV-2 infection risk and COVID-19 severity?. *Cell Biochemistry and Function*.

[124] Rhodes J. M., Subramanian S., Laird E., Griffin G., Kenny R. A. (2020). Perspective: Vitamin D deficiency and COVID-19 severity - plausibly linked by latitude, ethnicity, impacts on cytokines, ACE2 and thrombosis. *Journal of Internal Medicine*.

[125] Jain S. K., Parsanathan R., Levine S. N., Bocchini J. A., Holick M. F., Vanchiere J. A. (2020). The potential link between inherited G6PD deficiency, oxidative stress, and vitamin D deficiency and the racial inequities in mortality associated with COVID-19. *Free Radical Biology and Medicine*.

[126] Burton G. W., Ingold K. U. (1989). Vitamin E as an in vitro and in vivo antioxidant. *Annals of the New York Academy of Sciences*.

[127] Jovic T. H., Ali S. R., Ibrahim N. (2020). Could vitamins help in the fight against COVID-19?. *Nutrients*.

[128] Traber M. G., Atkinson J. (2007). Vitamin E, antioxidant and nothing more. *Free radical biology and medicine*.

[129] Sülzle A., Hirche F., Eder K. (2004). Thermally oxidized dietary fat upregulates the expression of target genes of PPAR*α* in rat liver. *The Journal of nutrition*.

[130] Lee G. Y., Han S. N. (2018). The role of vitamin E in immunity. *Nutrients*.

[131] Galabov A. S., Mileva M., Simeonova L., Gegova G. (2015). Combination activity of neuraminidase inhibitor oseltamivir and *α*-tocopherol in influenza virus A (H3N2) infection in mice. *Antiviral Chemistry and Chemotherapy*.

[132] Kim Y., Kim H., Bae S. (2013). Vitamin C is an essential factor on the anti-viral immune responses through the production of interferon-*α*/*β* at the initial stage of influenza A virus (H3N2) infection. *Immune network*.

[133] Bonnefont-Rousselot D. (2016). Resveratrol and cardiovascular diseases. *Nutrients*.

[134] Zhai T., Li S., Hu W., Li D., Leng S. (2018). Potential micronutrients and phytochemicals against the pathogenesis of chronic obstructive pulmonary disease and lung cancer. *Nutrients*.

[135] Rauf A., Imran M., Butt M. S., Nadeem M., Peters D. G., Mubarak M. S. (2018). Resveratrol as an anti-cancer agent: a review. *Critical Reviews in Food Science and Nutrition*.

[136] Tiao M. M., Lin Y. J., Yu H. R. (2018). Resveratrol ameliorates maternal and post-weaning high-fat diet-induced nonalcoholic fatty liver disease via renin-angiotensin system. *Lipids in Health and Disease*.

[137] Moran C. S., Biros E., Krishna S. M. (2017). Resveratrol inhibits growth of experimental abdominal aortic aneurysm associated with upregulation of angiotensin-converting enzyme 2. *Arteriosclerosis, Thrombosis, and Vascular Biology*.

[138] Palamara A. T., Nencioni L., Aquilano K. (2005). Inhibition of influenza A virus replication by resveratrol. *The Journal of Infectious Diseases*.

[139] Cai J., Chen Y., Seth S., Furukawa S., Compans R. W., Jones D. P. (2003). Inhibition of influenza infection by glutathione. *Free Radical Biology & Medicine*.

[140] Ahmed N., Chakrabarty A., Guengerich F. P., Chowdhury G. (2020). Protective role of glutathione against peroxynitrite-mediated DNA damage during acute inflammation. *Chemical Research in Toxicology*.

[141] Sies H., Parnham M. J. (2020). Potential therapeutic use of ebselen for COVID-19 and other respiratory viral infections. *Free Radical Biology and Medicine*.

[142] Duong C., Seow H. J., Bozinovski S., Crack P. J., Anderson G. P., Vlahos R. (2010). Glutathione peroxidase-1 protects against cigarette smoke-induced lung inflammation in mice. *American Journal of Physiology-Lung Cellular and Molecular Physiology*.

[143] Yatmaz S., Seow H. J., Gualano R. C. (2013). Glutathione peroxidase-1 reduces influenza A virus–induced lung inflammation. *American journal of respiratory cell and molecular biology*.

[144] Dröge W., Breitkreutz R. (2000). Glutathione and immune function. *The Proceedings of the Nutrition Society*.

[145] Liu X., Wang N., Fan S. (2016). The citrus flavonoid naringenin confers protection in a murine endotoxaemia model through AMPK-ATF3-dependent negative regulation of the TLR4 signalling pathway. *Scientific Reports*.

[146] Yu D. H., Ma C. H., Yue Z. Q., Yao X., Mao C. M. (2015). Protective effect of naringenin against lipopolysaccharide-induced injury in normal human bronchial epithelium via suppression of MAPK signaling. *Inflammation*.

[147] Podder B., Song H. Y., Kim Y. S. (2014). Naringenin exerts cytoprotective effect against paraquat-induced toxicity in human bronchial epithelial BEAS-2B cells through NRF2 activation. *Journal of Microbiology and Biotechnology*.

[148] De Flora S., Grassi C., Carati L. (1997). Attenuation of influenza-like symptomatology and improvement of cell-mediated immunity with long-term N-acetylcysteine treatment. *The European Respiratory Journal*.

[149] Khan N. A., Singla M., Samal S., Lodha R., Medigeshi G. R. (2020). Respiratory syncytial virus-induced oxidative stress leads to an increase in labile zinc pools in lung epithelial cells. *mSphere*.

[150] Shi X., Shi Z., Huang H., Zhu H., Zhou P., Ju D. (2014). Ability of recombinant human catalase to suppress inflammation of the murine lung induced by influenza A. *Inflammation*.

[151] Shi X. L., Shi Z. H., Huang H., Zhu H. G., Zhou P., Ju D. (2010). Therapeutic effect of recombinant human catalase on H1N1 influenza-induced pneumonia in mice. *Inflammation*.

[152] Xu J., Li T., Wu H., Xu T. (2012). Role of thioredoxin in lung disease. *Pulmonary pharmacology & therapeutics*.

[153] Moi P., Chan K., Asunis I., Cao A., Kan Y. W. (1994). Isolation of NF-E2-related factor 2 (Nrf2), a NF-E2-like basic leucine zipper transcriptional activator that binds to the tandem NF-E2/AP1 repeat of the beta-globin locus control region. *Proceedings of the National Academy of Sciences*.

[154] Callister M. E., Burke-Gaffney A., Quinlan G. J. (2006). Extracellular thioredoxin levels are increased in patients with acute lung injury. *Thorax*.

[155] Yashiro M., Tsukahara H., Matsukawa A. (2013). Redox-active protein thioredoxin-1 administration ameliorates influenza A virus (H1N1)-induced acute lung injury in mice. *Critical care medicine*.

[156] Tanaka R., Ishima Y., Enoki Y. (2014). Therapeutic impact of human serum albumin–thioredoxin fusion protein on influenza virus-induced lung injury mice. *Frontiers in immunology*.

[157] Okada K., Fukui M., Zhu B.-T. (2016). Protein disulfide isomerase mediates glutathione depletion-induced cytotoxicity. *Biochemical and biophysical research communications*.

[158] Alhammad R., Khunchai S., Tongmuang N. (2020). Protein disulfide isomerase A1 regulates breast cancer cell immunorecognition in a manner dependent on redox state. *Oncology Reports*.

[159] Lee T. H., Yeh C. F., Lee Y. T. (2020). Fibroblast-enriched endoplasmic reticulum protein TXNDC5 promotes pulmonary fibrosis by augmenting TGF*β* signaling through TGFBR1 stabilization. *Nature Communications*.

[160] Chamberlain N., Korwin-Mihavics B. R., Nakada E. M. (2019). Lung epithelial protein disulfide isomerase A3 (PDIA3) plays an important role in influenza infection, inflammation, and airway mechanics. *Redox biology*.

